# Characteristics of Registered Studies of Chimeric Antigen Receptor Therapies: A Systematic Review

**DOI:** 10.1001/jamanetworkopen.2021.15668

**Published:** 2021-07-08

**Authors:** Rahul Banerjee, Vinay Prasad

**Affiliations:** 1 Division of Hematology/Oncology, Department of Medicine, University of California, San Francisco; 2 Department of Epidemiology and Biostatistics, San Francisco General Hospital, San Francisco, California; 3 Department of Hematology Oncology, University of California, San Francisco

## Abstract

**Importance:**

Hundreds of chimeric antigen receptor (CAR) therapies are under investigation for hematologic malignant cancers and solid malignant tumors. As the field of modern CAR therapy enters its second decade, clinical trials that demonstrate the efficacy of CAR therapies using randomized clinical trials (RCTs) and/or investigate methods to optimize patient outcomes with commercially available CAR therapies are increasingly important.

**Objective:**

To analyze the landscape of registered CAR-related trials with dual focuses on trial methods and intent.

**Evidence Review:**

This systematic review identified 1304 ongoing or upcoming CAR-related trials registered at ClinicalTrials.gov as of December 22, 2020, and excluded 513 trials that did not pertain to cell-based therapy. Both CAR-related and trial-related variables, including target antigens and countries of origin, were recorded. Trials were categorized as non-RCTs that compared CAR with non-CAR therapies or RCTs in which every arm received CAR therapy. Trial intent was separately categorized as demonstrating the efficacy of a CAR therapy, optimizing patient outcomes with established CAR therapies using adjunctive non-CAR modalities, or miscellaneous.

**Findings:**

Of 778 relevant trials, 587 (75%) involved blood cancers, whereas 182 (23%) involved solid tumor cancers; the remaining 9 (1%) involved nonmalignant diseases. A total of 433 trials (56%) were from China and 288 from the US (37%). Ten RCTs (1%) compared CAR therapies with non-CAR therapies, including phase 3 RCTs for 4 of 5 CAR therapies (80%) that are currently commercially available. Twenty-eight studies (4%) sought to optimize outcomes with established CAR therapies using non-CAR drugs or radiotherapy, whereas 3 studies (0.4%) sought to optimize supportive care during CAR therapy.

**Conclusions and Relevance:**

This systematic review found that randomized and optimization-focused trials are comparatively rare within the landscape of ongoing and upcoming CAR-related trials. As the field of modern CAR therapy enters its second decade, additional studies of these characteristics are necessary to strengthen the evidence base for CAR therapy and improve patient outcomes.

## Key Points

**Question:**

What percentage of trials involving chimeric antigen receptor (CAR) therapy are randomized clinical trials or seek to optimize outcomes with commercially available CAR therapies?

**Findings:**

In this systematic review of 778 CAR-related trials, 10 (1%) were randomized clinical trials that compared CAR therapies with non-CAR therapies. Twenty-eight trials (4%) sought to optimize the efficacy, safety, or postrelapse outcomes with existing CAR therapies.

**Meaning:**

As modern CAR therapy enters its second decade, this analysis of the CAR therapy landscape suggests that more trials are needed to convincingly demonstrate its efficacy and investigate strategies to improve its safety and efficacy.

## Teaser

This systematic review analyzes the characteristics of registered chimeric antigen receptor–related trials with dual focuses on trial methods and intent.

## Introduction

Since the initial studies^1,2^ of second-generation chimeric antigen receptor (CAR) therapies were published in 2011, development of this form of cellular therapy has advanced rapidly. As of April 2021, 4 CAR-transduced T-cell (CAR-T) therapies have been approved by the US Food and Drug Administration (FDA) for CD19-expressing hematologic malignant cancers: tisagenlecleucel, axicabtagene ciloleucel, brexucabtagene autoleucel, and lisocabtagene maraleucel. Idecabtagene vicleucel, a CAR-T therapy that targets B-cell maturation antigen (BCMA), has also recently gained approval for the treatment of multiple myeloma. Enthusiasm exists not only for these autologous CAR-T therapies but also for allogeneic strategies that do not require several weeks of manufacturing after being collected from the patient and CAR-transduced natural killer therapies, which may offer manufacturing and toxicity-related advantages.^3,4^ There is no doubt that innovations in bioengineering will redefine the science behind modern CAR therapy as the field advances past 2021 into its second decade.

Previous studies^5,6,7,8,9,10,11^ on the characteristics of CAR-T therapies have focused on technical CAR characteristics and trial countries of origin. To our knowledge, no umbrella review has examined the methods and thematic intent of CAR-related trials. From a methodologic perspective, randomized clinical trials (RCTs) comparing the efficacy of CAR therapies with standard of care (SOC) therapies are essential for decision-making by patients, physicians, payers, and regulatory authorities. From a thematic perspective, studies of strategies to improve the toxicity profile, real-world effectiveness, and patient experience are needed to expand the availability and accessibility of CAR therapies. Despite the importance of such investigations, we hypothesized that the proportion of trials investigating the efficacy of CAR therapies through RCTs or strategies to improve outcomes of existing CAR therapies through pharmacologic or nonpharmacologic tools would be below 5%. We thus sought to better quantify the characteristics of CAR trials through a systematic review of trials listed at ClinicalTrials.gov with dual focuses on trial methods and study intent.

## Methods

### Search Strategy and Selection Criteria

In this systematic review, we performed a nonpreregistered systematic analysis of all CAR-related trials registered by their sponsors at ClinicalTrials.gov, a registry of human trials maintained by the US government. We specifically searched ClinicalTrials.gov for trial descriptions that included the phrases *chimeric antigen receptor*, *CAR*, or *CAR-T* (without requiring whole-word or case-sensitive matches). Related but older terms, for example, *chimeric immunoreceptors* and *artificial T-cell receptors*, were automatically included in the search algorithm. Trials in any of the following recruitment categories were included: *not yet recruiting*; *recruiting*; *enrolling by invitation*; *active, not recruiting*; *suspended*; or *unknown status*. Given our forward-looking emphasis on CAR therapies in the coming decade, trials listed as *terminated*, *completed*, or *withdrawn* were excluded. This ClinicalTrials.gov query was performed on December 22, 2020; all further analyses detailed herein were conducted within the following month. This study followed the Preferred Reporting Items for Systematic Reviews and Meta-analyses (PRISMA) reporting guideline.^12^

**Figure 1.**
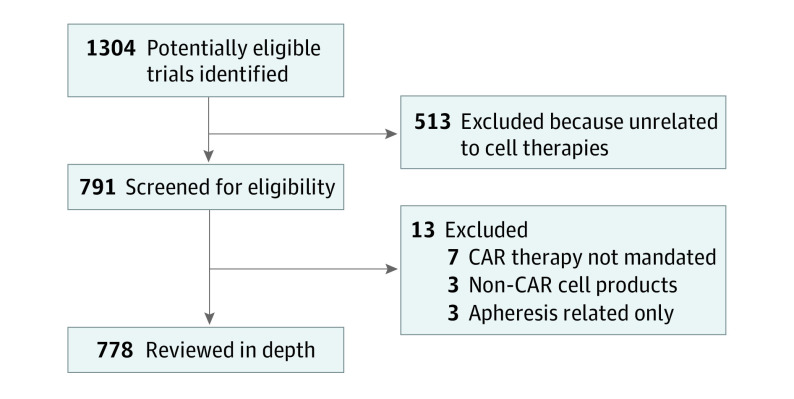
Flow Diagram of Included Trials CAR indicates chimeric antigen receptor.

We initially identified 1304 potentially eligible trials but excluded 513 trials that did not pertain to cell-based therapy. Of the 791 trials that were screened in full, 13 (2%) were excluded after in-depth reviews of their pertinence to this study (Figure 1). To eliminate trials not pertinent to CAR therapies, 1 author (R.B.) analyzed the titles and descriptions of each trial (as entered by study sponsors, all in English) on ClinicalTrials.gov. Unrelated trials were excluded, for example, trials referencing *car* as a reference to automobiles, *car* as a syllable within *carboplatin*, or *car* as an abbreviation for combined antiretroviral therapy. Of trials that referenced CAR therapies, trials that analyzed only pre-CAR apheresis or did not strictly involve CAR-engineered constructs (eg, trials of unmodified tumor-infiltrating lymphocytes) were excluded. We excluded therapeutic trials in which CAR therapy was optional off protocol; however, trials of treatment algorithms that formally incorporated CAR therapy were included. Studies that analyzed correlative end points or predictive assessments around the time of CAR therapy (eg, frailty assessments or short-term imaging findings) were also included. Gray-area trials were reviewed between authors until consensus was reached. Because of the heterogeneity of trials and (in many cases) paucity of information listed at ClinicalTrials.gov, quality appraisals of included trials were not conducted.

### Statistical Analysis

We sought to update the results of previous studies^5,6,7,8,9,10^ of the CAR trial landscape that have primarily focused on disease-related, CAR-related, and geographic factors. Thus, we extracted and verified the following fields from ClinicalTrials.gov records: (1) target antigen, with multitarget CAR products classified accordingly; (2) destination cell line, either autologous T cells vs allogeneic products (eg, allogeneic natural killer cells or universal T cells); and (3) trial country of origin, with multinational studies classified based on their first listed study center. We also collected planned sample sizes as reported by trial sponsors. Last, we categorized trials based on their patient population: hematologic malignant cancers, solid malignant tumors, and noncancer conditions (eg, CAR therapies for autoimmune diseases). The CAR-related trials enrolling patients with solid tumors or hematologic malignant cancers were classified as solid malignant tumor trials.

Next, we classified trials based on methodologic and thematic elements. First, we categorized trials based on their method of treatment arm allocation: (1) non-RCTs, including single-arm studies; (2) RCTs in which a CAR-containing arm was compared with at least 1 SOC arm that did not receive any CAR therapy; or (3) RCTs in which every arm received at least 1 CAR therapy. Second, we categorized trials into 1 of 3 domains based on their thematic intent: (1) demonstrating the efficacy of a CAR therapy, (2) optimizing outcomes of established CAR therapies, or (3) serving a miscellaneous intent. As detailed in the Box, efficacy-oriented trials included studies of novel CAR constructs or trials investigating new indications for established CAR therapies. In contrast, optimization-oriented trials applied to established CAR therapies and involved the addition of non-CAR adjunctive therapies (eg, drugs or radiotherapy), either during CAR therapy administration or at the time of relapse. We defined *established* CAR therapies as 1 of 5 products that have gained FDA approval as of April 2021: tisagenlecleucel, axicabtagene ciloleucel, brexucabtagene autoleucel, lisocabtagene maraleucel, and idecabtagene vicleucel. Of note, lisocabtagene maraleucel and idecabtagene vicleucel were not approved at the time of our data analysis in December 2020; however, we included them as *established* CAR therapies at the time given their imminently expected approvals.

Box. **Thematic Intents of CAR-Related Trials**

**Demonstrating the Efficacy of a CAR Therapy**

Investigating a novel CAR target or CAR construct
Validating the efficacy of an established CAR in a larger setting^a^
Expanding an established CAR beyond its FDA package insert, by investigating a new disease or by investigating nonapproved administration characteristics (including EAPs)^a^

**Optimizing the Outcomes of Established CAR Therapies**

Improving the efficacy of an established CAR by adding a separate non-CAR therapy as part of a prespecified protocol^a,b^
Improving the safety of an established CAR by adding a separate non-CAR therapy as part of a prespecified protocol^a,b^
Investigating strategies to treat patients with refractory or relapsed disease after CAR therapy (as a prespecified cohort)

**Serving a Miscellaneous Intent**

Bettering the field’s understanding of CAR functions or toxic effects through PRO assessments, imaging results, or laboratory specimens
Investigating supportive care strategies during CAR-based therapies

Abbreviations: CAR, chimeric antigen receptor; EAP, expanded access program; FDA, US Food and Drug Administration; PRO, patient-reported outcome.

^a^Established CARs were defined as CAR therapies that have gained FDA approval as of April 2021: tisagenlecleucel, axicabtagene ciloleucel, brexucabtagene autoleucel, lisocabtagene maraleucel, and idecabtagene vicleucel.

^b^Separate non-CAR therapies include other pharmacologic agents or radiotherapy. Supportive care strategies, for example, wearable patient devices or resources for psychosocial support, were included in the miscellaneous category.

All data were collected from existing ClinicalTrials.gov records, with manual cross-referencing against publicly available research abstracts (using Google Scholar searches) performed only if data for our analyses could not be elucidated directly from a trial’s ClinicalTrials.gov record. Study sponsors were not contacted as part of this review. Data were analyzed and visualized descriptively using Microsoft Excel (Microsoft Corp) and Stata (StataCorp LLC). Where appropriate, categorical variables were compared using Fisher exact tests, and nonparametric variables were compared using Wilcoxon rank-sum tests. Statistical significance was defined as a 2-sided *P* < .05.

## Results

Characteristics of the 778 trials included in this review are given in Table 1. Nine trials (1% of all trials) involved patients without cancer, of which 6 trials (67%) targeted infectious diseases and 3 (33%) targeted autoimmune diseases. Of 587 CAR trials that involved hematologic malignant cancers (75% of all trials), CD19 was targeted in 366 (62%) (including as part of a multiantigen-targeting approach in 74 trials), whereas BCMA was targeted in 86 (15%) (including as part of a multiantigen-targeting approach in 19 trials). Of 182 CAR trials that involved solid malignant tumors (23% of all trials), the most common targets were mesothelin (23 [13%]), members of the epidermal growth factor receptor family (14 [8%]), and the surface disialoganglioside GD2 (14 [8%]); however, 43 other CAR targets in various solid malignant tumors were reported in trials as well.

**Table 1.** Characteristics of CAR-Related Trials

Characteristic	No. (%) of trials^a^			
	Total (N = 778)	Hematologic (n = 587)	Solid (n = 182)	Noncancer (n = 9)
Type of target				
Single antigen	684 (88)	502 (86)	173 (95)	9 (100)
Multiple antigens	94 (12)	85 (14)	9 (5)	0
Type of cell				
Autologous T cell	715 (92)	538 (92)	170 (93)	7 (78)
Universal T cell	44 (6)	39 (7)	4 (2)	1 (11)
Natural killer cell	19 (2)	10 (2)	8 (4)	1 (11)
Country of origin				
China	433 (56)	322 (55)	105 (58)	6 (67)
US	288 (37)	220 (37)	65 (36)	3 (33)
European country	41 (5)	32 (5)	9 (5)	0
Other	16 (2)	13 (2)	3 (2)	0
Planned sample size^b^				
<20 patients	216 (28)	162 (28)	49 (27)	5 (56)
20-49 patients	335 (43)	241 (41)	92 (51)	2 (22)
50-99 patients	131 (17)	106 (18)	24 (13)	1 (11)
≥100 patients	90 (12)	72 (12)	17 (9)	1 (11)
Thematic objective				
Efficacy	732 (94)	542 (92)	181 (99)	9 (100)
Optimization	28 (4)	28 (5)	0	0
Miscellaneous	18 (2)	17 (3)	1 (1)	0
Randomization^c^				
Nonrandomized	753 (97)	571 (97)	176 (97)	6 (67)
SOC randomized	10 (1)	5 (1)	4 (2)	1 (11)
CAR randomized	15 (2)	11 (2)	2 (1)	2 (22)

Abbreviations: CAR, chimeric antigen receptor; SOC, standard of care.

^a^ Percentages may not total 100% because of rounding.

^b^ As reported by the trial sponsor (missing for 6 trials).

^c^ Nonrandomized trials did not use randomization. SOC randomized trials assigned patients randomly between at least 2 arms, with at least 1 arm receiving a control (non-CAR) therapy. CAR randomized trials assigned patients randomly between at least 2 arms, all of which received a CAR therapy.

**Figure 2.**
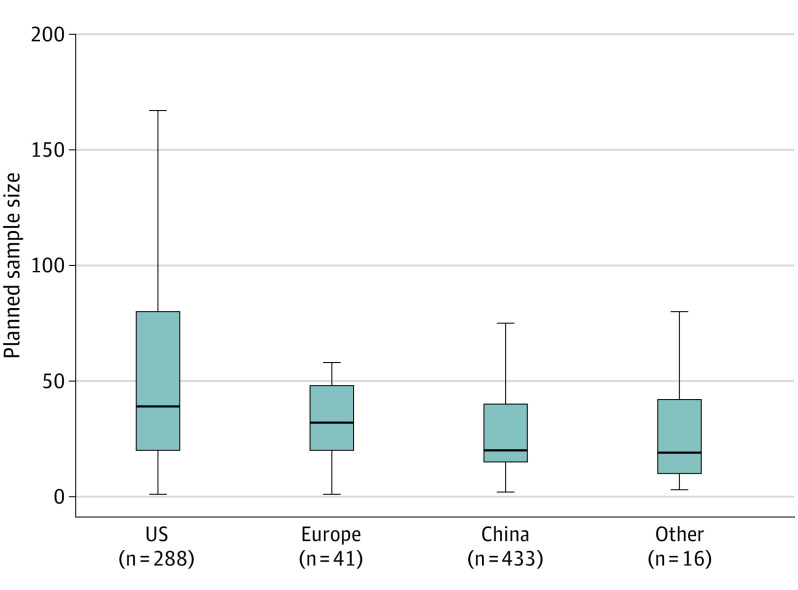
Box Charts of Planned Trial Sample Sizes Median sample sizes are shown. Outlier values are not shown. Error bars indicate interquartile ranges

Investigations of allogeneic CAR strategies constituted 8% of all trials, with similar distributions between blood cancers (50 [9%]) and solid tumor cancers (12 [7%], *P* = .53). With regard to country of origin, 433 CAR trials (56%) were based in China, whereas 288 (37%) were based in the US. As reported in Table 1, the distributions of countries of origin were similar between hematologic blood cancers and solid tumor cancers; US-based trials constituted 220 trials (37%) that involved blood cancers and 65 trials (36%) that involved solid tumor cancers (*P* = .93). Planned sample sizes were also similar: a median of 30 patients for hematologic malignant cancer trials vs a median of 27 patients for solid malignant tumor trials, with *P* = .28 by Wilcoxon rank-sum test. In an exploratory analysis summarized in Figure 2, we found that planned sample sizes were larger for studies based in the US than in China: the medians were 39 patients in the US vs 20 patients in China, with *P* < .01 by Wilcoxon rank-sum testing. Ninety trials (12% of all trials) reported planned sample sizes of 100 patients or higher. Of these 90 trials, 55 (56%) were based in the US, whereas 34 (38%) were based in China.

Our analysis of the trial methods found that 10 trials (1% of all analyzed trials) were RCTs specifically designed to compare a CAR therapy with a non-CAR therapy (generally SOC treatments but reportedly placebo in 1 trial). As described in Table 2,^13,14,15,16,17,18,19,20,21,22^ such RCTs have been registered for the following established CAR therapies: tisagenlecleucel (Tisagenlecleucel in Adult Patients With Aggressive B-cell Non-Hodgkin Lymphoma [BELINDA]^16^), axicabtagene ciloleucel (Efficacy of Axicabtagene Ciloleucel Compared to Standard of Care Therapy in Subjects With Relapsed/Refractory Diffuse Large B Cell Lymphoma [ZUMA-7]^14^), lisocabtagene maraleucel (A Study to Compare the Efficacy and Safety of JCAR017 to Standard of Care in Adult Subjects With High-risk, Transplant-eligible Relapsed or Refractory Aggressive B-cell Non-Hodgkin Lymphomas [TRANSFORM] phase 3^17^), and idecabtagene vicleucel (Efficacy and Safety Study of bb2121 Vs Standard Regimens in Subjects With Relapsed and Refractory Multiple Myeloma (RRMM) [KarMMa-3]^18^). We were unable to find any ongoing or planned RCTs for brexucabtagene autoleucel, a CAR-T product that received FDA approval for the treatment of relapsed or refractory mantle cell lymphoma in July 2020. However, we identified a phase 3 RCT (A Study Comparing JNJ-68284528, a CAR-T Therapy Directed Against B-cell Maturation Antigen [BCMA], vs Pomalidomide, Bortezomib and Dexamethasone [PVd] or Daratumumab, Pomalidomide and Dexamethasone [DPd] in Participants With Relapsed and Lenalidomide-Refractory Multiple Myeloma [CARTITUDE-4]^22^) of ciltacabtagene autoleucel, a BCMA-targeted CAR-T therapy for which an FDA Biologics License Application has recently been initiated.^23^ As described in eTable 2 in the Supplement, 12 trials (2% of all trials) reported the use of randomization between arms in which all patients received CAR therapies. However, all these trials were phase 1 or 2 studies that involved experimental CAR constructs; there were no randomized head-to-head comparisons of established CAR therapies.

**Table 2.** Randomized Controlled Trials of CAR Therapies (Compared With at Least 1 SOC Therapy)

Trial	Indication	CAR arm	SOC arm(s)
A New EBV Related Technologies of T Cells in Treating Malignant Tumors and Clinical Application^13^ (phase 1/2, n = 20)	EBV-positive NPC	LMP1-directed (experimental)	Placebo (no further information available)
Efficacy of Axicabtagene Ciloleucel Compared to Standard of Care Therapy in Subjects With Relapsed/Refractory Diffuse Large B Cell Lymphoma^14^ (phase 3, n = 350)	R/R DLBCL	CD19-directed (axicabtagene ciloleucel)	Salvage chemotherapy, ASCT, or SOC
Anti-MUC1 CAR T Cells and PD-1 Knockout Engineered T Cells for NSCLC^15^ (phase 1/2, n = 60)	Advanced NSCLC	MUC1-directed (experimental)	Several arms, including pembrolizumab
Tisagenlecleucel in Adult Patients With Aggressive B-cell Non-Hodgkin Lymphoma^16^ (phase 3, n = 318)	Aggressive R/R NHL	CD19-directed (tisagenlecleucel)	Salvage chemotherapy, ASCT, or SOC
A Study to Compare the Efficacy and Safety of JCAR017 to Standard of Care in Adult Subjects With High-risk, Transplant-eligible Relapsed or Refractory Aggressive B-cell Non-Hodgkin Lymphomas^17^ (phase 3, n = 182)	Aggressive R/R NHL	CD19-directed (lisocabtagene maraleucel)	Salvage chemotherapy, ASCT, or SOC
Efficacy and Safety Study of bb2121 Vs Standard Regimens in Subjects With Relapsed and Refractory Multiple Myeloma (RRMM)^18^ (phase 3, n = 381)^a^	R/R MM	BCMA-directed (idecabtagene vicleucel)	SOC
Effect of Chidamide Combined With CAT-T or TCR-T Cell Therapy on HIV-1 Latent Reservoir^19^ (phase 1, n = 40)	HIV	Chidamide plus gp120-directed (experimental)	HAART
Study of Anti-CEA CAR-T + Chemotherapy VS Chemotherapy Alone in Patients With CEA+Pancreatic Cancer & Liver Metastases^20^ (phase 2B, n = 167)	Pancreatic cancer	CEA-targeted intrahepatic infusions (experimental)	Several arms, including chemotherapy alone
B7-H3 CAR-T for Recurrent or Refractory Glioblastoma^21^ (phase 1/2, n = 40)	R/R GBM	B7-H3–targeted (experimental)	Temozolomide (also given to CAR arm)
A Study Comparing JNJ-68284528, a CAR-T Therapy Directed Against B-cell Maturation Antigen (BCMA), Vs Pomalidomide, Bortezomib and Dexamethasone (PVd) or Daratumumab, Pomalidomide and Dexamethasone (DPd) in Participants With Relapsed and Lenalidomide-Refractory Multiple Myeloma^22^ (phase 3, n = 400)	R/R MM	BCMA-directed (experimental)^b^	SOC

Abbreviations: ASCT, autologous stem cell transplantation; BCMA, B-cell maturation antigen; B7-H3, B7 protein homologue 3; CAR, chimeric antigen receptor; CEA, carcinoembryonic antigen; DLBCL, diffuse large B-cell lymphoma; EBV, Epstein-Barr virus; GBM, glioblastoma multiforme; gp120, envelope glycoprotein 120; HAART, highly active antiretroviral therapy; LMP1, latent membrane protein 1; MM, multiple myeloma; MUC1, cell surface–associated mucin 1; NHL, non-Hodgkin lymphoma; NPC, nasopharyngeal carcinoma; NSCLC, non–small cell lung cancer; R/R, relapsed/refractory; SOC, standard of care.

^a^ Trial conducted with 2:1 randomization for the CAR arm vs SOC arm. Other trials report 1:1 randomization or do not list their randomization algorithm.

^b^ As of April 2021, this product (also known as ciltacabtagene autoleucel) had not yet received FDA approval.

Our analysis of trial intent found that 732 trials (94%) fell into the domain of investigating CAR efficacy, including 707 trials of novel CAR constructs and 25 trials seeking to expand established CAR therapies beyond their approved indications (and the remainder of trials defining expanded access programs). As indicated in eTable 1 in the Supplement, 28 studies (4% of all trials) fell into the domain of optimizing outcomes with established CAR therapies using pharmacologic or radiotherapy additions. These strategies were under investigation to improve CAR efficacy (n = 9), improve CAR safety (n = 11), or identify postCAR treatment strategies (n = 8). Of these 28 studies, immune checkpoint inhibitors or Bruton tyrosine kinase inhibitors were under investigation in 5 trials each (18%). Similarly, the interleukin 1 receptor antagonist anakinra was under investigation in 5 trials. Last, as also indicated in eTable 1 in the Supplement, 3 of the 18 *miscellaneous* trials investigated supportive-care strategies, such as mobile apps or psychosocial counseling, during commercially available CAR-T therapy.

## Discussion

To our knowledge, this systematic review is the first effort to classify the characteristics of CAR-related trials with respect to methods and intent. As expected, the study found an impressive diversity of CAR-related research being conducted or planned, including studies of allogenic products and even of CAR therapies for nonmalignant conditions. However, fewer than 5% of registered trials sought to address the important concerns about the efficacy of CAR therapies as evidenced through RCTs or strategies to improve patient outcomes or experiences with CAR therapies.

Specifically, only 1% of trials constituted RCTs of CAR therapies vs SOC therapies (the criterion standard of clinical research in oncology),^24^ and this analysis was unable to identify any ongoing or planned RCTs for the FDA-approved CAR-T therapy brexucabtagene autoleucel. Furthermore, although CAR-T therapies have been commercially available on several continents since as early as 2017,^11^ only 4% of registered studies sought to build on previous results to optimize outcomes with these products in a scientific manner. As the field of modern CAR therapy enters its second decade, many more trials with these types of study characteristics should become available to make CAR therapy safer, more effective, more evidence based, and ultimately more widely available.

The analyses of CAR targets and trial countries of origin are consistent with previous studies.^5,7^ Compared with 2 analyses^5,7^ of CAR-related trials registered at ClinicalTrials.gov conducted in December 2016 (113 trials) and December 2017 (289 trials), the current analysis from December 2020 (778 trials) demonstrates a similar preponderance of trials registered in China or the US (with more trials based in China but larger planned sample sizes in US studies).^5,7^ Compared with the 2017 analysis,^7^ CD19 and BCMA continue to remain the most common targets, whereas allogeneic cell lines continue to comprise less than 10% of all trials. However, CAR therapies that target multiple antigens synchronously (eg, bispecific CAR-T cells or simultaneous infusions of different CAR products) comprised only 4% of trials in the 2017 analysis; in this updated analysis, such therapies represented 12% of trials.^7^ This finding is consistent with a recent analysis^11^ of CAR-related trials that identified bispecific CAR constructs as an emerging cluster of bioengineering innovation with the potential to improve CAR affinity for tumor cells and decrease clinical rates of relapse.

The current analysis of methods and intent reveals 2 main conclusions. First, randomization is rare in CAR-related studies. This analysis found only 10 registered RCTs comparing CAR therapies with non-CAR therapies; however, these 10 studies ranged from 40-patient phase 1 studies of solid oncologic tumors to 400-patient phase 3 studies of hematologic cancers. For the 3 established CD19-directed CAR therapies with FDA approval for aggressive B-cell lymphomas in the third-line setting (tisagenlecleucel, axicabtagene ciloleucel, and lisocabtagene maraleucel), RCTs comparing these therapies with SOC therapy in the second-line setting are being conducted through the BELINDA study,^16^ ZUMA-7 study,^14^ and the TRANSFORM phase 3 study.^17^ Equipoise between CAR-T therapy and SOC chemotherapies certainly is more feasible here than in the third-line setting for patients with chemorefractory disease. In contrast, brexucabtagene autoleucel, another CD19-directed CAR-T therapy that received accelerated FDA approval in July 2020 for the treatment of relapsed or refractory mantle cell lymphoma, did not have an analogous phase 3 RCT registered at ClinicalΤrials.gov as of our data cutoff.

A second finding of this study is the paucity of studies of aftermarket CAR products that seek to enhance the safety profile, long-term efficacy, or patient experience with commercial CAR therapies. Aftermarket car products in the automotive industry serve such an optimization-focused purpose; evidently, however, few such aftermarket trials exist in the world of CAR products. As an example, tocilizumab is a mainstay of management for cytokine release syndrome (CRS) after CAR-T therapy and has received FDA approval for this indication. However, this approval of tocilizumab was based on retrospective pooled analyses of single-arm studies of CAR-T therapies rather than a tocilizumab-focused study to optimize its dose and timing.^25^ In addition, although tocilizumab’s FDA approval recommends its use for severe or life-threatening CRS, almost half of patients with early-grade CRS receive tocilizumab in the real-world setting.^26^ Similarly, although the FDA package inserts for tisagenlecleucel and axicabtagene ciloleucel recommend tocilizumab only for severe or life-threatening CRS (in line with the package insert for tocilizumab), the corresponding package inserts for newer CAR-T therapies also recommend tocilizumab for persistent or early-onset grade 1 CRS. These evolving discrepancies highlight the need for future studies to optimize supportive care with CAR-T therapy in a more systematic manner for tocilizumab and other toxicity mitigation strategies.

### Limitations

This study has limitations. First, the study relied on data provided by sponsors to ClinicalTrials.gov. Although all information was provided in English, language-related or typographic errors may have led to corresponding errors in this analysis. Second, target antigens and allogeneic cell lines (eg, universal CAR-T cells) were recorded only if these items were explicitly mentioned in trial descriptions; thus, the proportions of trials targeting multiple antigens or using allogeneic cell lines may have been underestimated. Third, given the focus on ongoing and upcoming studies, studies that were listed as having been completed were excluded; however, a subset of these studies may be reopened in the future with additional cohorts that modify their methods or intent. Fourth, characterizing the methods or intent of trials inherently has some element of subjectivity, for example, how established CAR-T therapies were defined and whether an expanded access program qualified as an efficacy-oriented trial or an optimization-oriented trial (this study chose the former). However, the data unequivocally suggest that efficacy-oriented studies remain the most common type of CAR-related trial regardless of the nuances of thematic classification.

## Conclusions

This systematic review of ongoing and upcoming trials assessing CAR therapies found interest in a variety of targets and cell lines across malignant and even nonmalignant diseases. Although a substantial percentage of trials (94%) sought to establish the efficacy of these products, only 1% of studies randomized CAR therapies against the SOC. Similarly, only 4% of trials sought to optimize efficacy-related, safety-related, or relapse-related outcomes with CAR therapies that have already been approved. It is hoped that larger numbers of RCTs of CAR-T products will be seen in the coming decade, including head-to-head comparisons of next-generation CAR products vs established CAR products. Furthermore, multicenter collaborations (eg, the US Lymphoma CAR-T Consortium)^27^ may rapidly accrue patients to investigate strategies that incrementally change how commercially available CAR therapies are chosen and administered. These types of trials are particularly important as modern CAR therapy is increasingly incorporated in clinical practice. Future trials will strengthen the evidence base for CAR therapies and, in so doing, improve both physician and patient experience with CAR therapies.

## Notes

Supplement. **eTable 1.** Trials Seeking to Optimize Established CAR Therapies **eTable 2.** Randomized Trials Comparing CAR Therapies Against Other CAR Therapies
