# Supplementary material for: Characteristics of Registered Studies of Chimeric Antigen Receptor Therapies: A Systematic Review
Source: JAMA Netw Open. 2021 Jul 8;4(7):e2115668. doi: 10.1001/jamanetworkopen.2021.15668 (PMC8267610; doi:10.1001/jamanetworkopen.2021.15668)
Supplement: Supplement. — eTable 1. Trials Seeking to Optimize Established CAR Therapies eTable 2. Randomized Trials Comparing CAR Therapies Against Other CAR Therapies [file jamanetwopen-e2115668-s001.pdf]

Supplementary Online Content

Banerjee R, Prasad V. Characteristics of registered studies of chimeric antigen receptor therapies: a systematic review. *JAMA Netw Open*. 2021;4(7):e2115668. doi:10.1001/jamanetworkopen.2021.15668

eTable 1. Trials Seeking to Optimize Established CAR Therapies

eTable 2. Randomized Trials Comparing CAR Therapies Against Other CAR Therapies

This supplementary material has been provided by the authors to give readers additional information about their work.

© 2021 Banerjee R et al. *JAMA Network Open.*

eTable 1. Trials Seeking to Optimize Established CAR Therapies

Identifier	Product(s)	Intervention
Optimizing the efficacy of established CAR therapies		
**Identifier**	**Product(s)**	**Intervention**
NCT02926833	Axi-cel	Atezolizumab
NCT03310619	Liso-cel	Durvalumab, avadomide/iberdomide (IMiDs), or ibrutinib
NCT03331198	Liso-cel	Ibrutinib
NCT03630159	Tisa-cel	Pembrolizumab
NCT03704298	Axi-cel	Utomilumab (4-1BB agonist)
NCT03876028	Tisa-cel	Ibrutinib
NCT04002401	Axi-cel	Rituximab
NCT04234061	Tisa-cel	Ibrutinib
NCT04257578	Axi-cel	Acalabrutinib
Optimizing the safety of established CAR therapies		
**Identifier**	**Product(s)**	**Intervention**
NCT02906371	Tisa-cel	Early dosing of tocilizumab
NCT03954106	Axi-cel	Defibrotide
NCT04048434	*Not specified*	Extracorporeal cytokine adsorption
NCT04071366	Tisa-cel, axi-cel	Itacitinib (Janus kinase inhibitor)
NCT04148430	*Not specified*	Anakinra
NCT04150913	Axi-cel	Anakinra
NCT04205838	Axi-cel	Anakinra
NCT04314843	Axi-cel	Lenzilumab (GM-CSF antagonist)
NCT04359784	Axi-cel	Anakinra
NCT04432506	Axi-cel	Anakinra
NCT04514029	Axi-cel	Intrathecal dexamethasone and oral simvastatin
Identifying strategies for R/R disease after commercial CAR therapies		
**Identifier**	**Product(s)**	**Intervention**
NCT02290951	*Not specified*	REGN1979 (CD20xCD3 bispecific antibody)
NCT02650999	Tisa-cel	Pembrolizumab
NCT03648372	*Not specified*	TAK-981(inhibitor of ubiquitin-like pathway)
NCT04074330	*Not specified*	TAK-981 plus rituximab
NCT04136756	*Not specified*	NKTR-255 (interleukin-15 receptor agonist)
NCT04205409	*Not specified*	Nivolumab
NCT04473937	*Not specified*	Radiotherapy
NCT04601831	*Not specified*	Radiotherapy
Improving supportive care with established CAR therapies		
**Identifier**	**Product(s)**	**Intervention**
NCT04051216	*Not specified*	Mobile tablet app, wearable physical activity monitoring
NCT04280133	*Not specified*	Educational video about CAR therapies
NCT04390542	*Not specified*	Psychoeducational intervention for patients' caregivers

The putative mechanism of drugs without current indications are shown in parentheses.

Abbreviations: CAR, chimeric antigen receptor; IMiD, immunomodulatory imide drugs; GM-CSF, granulocyte-macrophage colony-stimulating factor; R/R, relapsed/refractory.

© 2021 Banerjee R et al. *JAMA Network Open.*

eTable 2. Randomized Trials Comparing CAR Therapies Against Other CAR Therapies*

Trial details	Indication	First arm†	Other arm(s)
NCT02992834 (Phase 1, *n* = 10)	NHL	CAR expansion with IL-2 stimulation	CAR expansion with IL-7/IL-15 stimulation
NCT03275493 (Phase 1/2, *n* = 40)	B-ALL	CAR with “CRS suppression technology”	CAR without this “technology”
NCT03549442 (Phase 1, *n* = 40)	MM	Two CARs (one against CD19, one against BCMA)	One CAR targeting BCMA
NCT03617198 (Phase 1, *n* = 12)	HIV	HAART interruption 1 day after CAR infusion	HAART interruption after 8 weeks
NCT03706326 (Phase 1, *n* = 20)	Esophageal cancer	MUC-1 CARs with PD-1 knockout	MUC-1 CARs alone, or PD-1 knockout alone
NCT03840317 (Phase 1, *n* = 20)	B-ALL	CAR with putatively lower cytokine secretion	CAR without this cytokine-related feature
NCT03943472 (Phase 1, *n* = 10)	MM	CAR against BCMA with “immune inhibitors”	CAR against BCMA without any additions
NCT04003649 (Phase 1, *n* = 60)	GBM	CAR against IL-13 with nivolumab and ipilimumab	CAR against IL-13 with ipilimumab alone
NCT04324996 (Phase 1/2, *n* = 90)	COVID-19	CAR-NK cells secreting IL-15, anti-GM-CSF	CAR-NK cells targeting ACE2
NCT04534634 (Phase 2, *n* = 60)	B-ALL	CAR cells given alongside interferon alpha	CAR cells given without interferon alpha
NCT04553393 (Phase 1/2, *n* = 80)	NHL	CARs with chidamide and decitabine	CARs with chidamide or decitabine alone
NCT04603872 (Phase 1, *n* = 120)	B-ALL, NHL, MM	CARs with dasatinib	CARs without dasatinib

Data are derived from clinicaltrials.gov records, including information in quotations.

* 3 studies not shown: NCT04048434 (randomized study of a cytokine adsorption technique among patients who develop CRS), NCT04280133 (randomized study of a supportive-care video during CAR-T therapy), or NCT04390542 (randomized study of a psychosocial intervention for caregivers of CAR-T therapy recipients).

† Defined as the more complex of multi-arm studies or, if both arms were equally complex in design, the first listed arm.

Abbreviations: CAR, chimeric antigen receptor; NHL, non-Hodgkin’s lymphoma; B-ALL, B-cell acute lymphoblastic leukemia; CRS, cytokine release syndrome; MM, multiple myeloma; HIV, human immunodeficiency virus; HAART, highly active anti-retroviral therapy; MUC1, cell-surface-associated mucin 1; PD-1, programmed death receptor 1; GBM, glioblastoma multiforme; COVID-19, Coronavirus Disease 2019; CAR-NK, CAR-transduced Natural Killer cells; GM-CSF, granulocyte-macrophage colony-stimulating factor.

© 2021 Banerjee R et al. *JAMA Network Open.*
